# Studies on antidyslipidemic effects of *Morinda citrifolia *(Noni) fruit, leaves and root extracts

**DOI:** 10.1186/1476-511X-9-88

**Published:** 2010-08-20

**Authors:** Saf-ur Rehman Mandukhail, Nauman Aziz, Anwarul-Hassan Gilani

**Affiliations:** 1 Natural Product Research Division, Department of Biological and Biomedical Sciences, Aga Khan University Medical College, Karachi 74800, Pakistan; 2 Department of Pharmacy, University of Baluchistan, Sariab Road, Quetta, Pakistan; 3 King Saud University, Riyadh, Saudi Arabia

## Abstract

**Background:**

The objective of present study was to provide the pharmacological basis for the medicinal use of *Morinda citrifolia *Linn in dyslipidemia using the aqueous-ethanolic extracts of its fruits (Mc.Cr.F), leaves (Mc.Cr.L) and roots (Mc.Cr.R).

**Results:**

Mc.Cr.F, Mc.Cr.L and Mc.Cr.R showed antidyslipidemic effects in both triton (WR-1339) and high fat diet-induced dyslipidemic rat models to variable extents. All three extracts caused reduction in total cholesterol and triglyceride levels in triton-induced dyslipidemia. In high fat diet-induced dyslipidemia all these extracts caused significant reduction in total cholesterol, triglyceride, low density lipoprotein-cholesterol (LDL-C), atherogenic index and TC/HDL ratio. Mc.Cr.R extract also caused increase in high density lipoprotein-cholesterol (HDL-C). The Mc.Cr.L and Mc.Cr.R reduced gain in body weight with a reduction in daily diet consumption but Mc.Cr.F had no effect on body weight and daily diet consumption.

**Conclusions:**

These data indicate that the antidyslipidemic effect of the plant extracts was meditated through the inhibition of biosynthesis, absorption and secretion of lipids. This may be possibly due partly to the presence of antioxidant constituents in this plant. Therefore, this study rationalizes the medicinal use of *Morinda citrifolia *in dyslipidemia.

## Background

Dyslipidemia is an independent and modifiable risk factor for cardiovascular diseases. Its prevalence is growing not only in developed countries but also in developing countries [1]. Treatment of dyslipidemia reduces cardiovascular events [2]. The modern pharmacological therapy for abnormal lipids is effective but is costly and associated with side-effects [3] leading to patient incompliance. Therefore, alternative therapies particularly, herbal based are being explored. *Morinda citrifolia *Linn (Fam. Rubiaceae) is commonly known as Noni. Different parts of the plant including fruit, leaves, root, stem and bark are used in folk medicine in Polynesia, Tahiti, Southeast Asia, Australia and Hawaii. It has been shown that these are effective against minimizing the symptoms of life style-related diseases such as atherosclerosis [4], hypertension [5] and other vascular disorders [4], stroke [6], diabetes and cancer [7]. Furthermore, Noni juice, a popular beverage is known to contain some antioxidative and anti-inflammatory ingredients [5].

*Morinda citrifolia*, has been reported to possess vasodilatory [8,9], antioxidant [10] antitumor [11] and Angiotensin Converting Enzyme inhibitor activities[12]. It is an edible plant and its fruit juice is a popular drink. Almost all parts of this plant have some medicinal value and have been widely studied phytochemically [see additional file 1].

Recently we have reported that antispasmodic and vasodilatory activities of *Morinda citrifolia *root extract are mediated through blockade of voltage-dependent calcium channels [9]. However, the plant is not widely studied for its antidyslipidemic effects except a preliminary report [13]. Therefore, the objective of the present study was to investigate the antidyslipidemic effect of *Morinda citrifolia *in Triton WR 1339 and high fat diet-induced dyslipidemia rat models to rationalize its medicinal use in cardiovascular disorders.

## Materials and methods

### Plant material

The vacuum dried 70% aqueous-ethanolic extract of *Morinda citrifolia *fruit, leaves and roots were gifted by the Sami Labs Limited 19/1, 19/2, 1st Main II Phase, Peenya Industrial Area, Bangalor-560 058, India. The extracts were prepared by following procedure as described by the manufacturer. Dried leaves, fruit and roots of *Morinda citrifolia *were procured from a reputed herb supplier in Southern India. The leaves, fruits and roots were chopped and ground by hammer mill and passed through 20 mesh screen. The powder (12 Kg) was extracted with 70% ethanol (48 L) at 70°C for 3 hours and filtered. The procedure repeated twice and all filtrates combined and evaporated under vacuum. The dried extracts were packed in polythene bags with nitrogen purge.

### Drugs and standards

The following reference chemicals were obtained from the source specified: Triton (tyloxapol), Cholic acid, cholesterol (Sigma Chemical Company, ST Louis, MO, USA) and diethylether (Sigma-Aldrich Chemie GmbH, Germany). Commercial Randox diagnostic kits were used for serum analyses of total cholesterol (TC), triglyceride (TG), high density lipoprotein-cholesterol (HDL-C) and glucose level (Randox Laboratories Ltd., Co. Antrim, UK). All chemicals used were of the highest purity grade.

### Animals

Sprague-Dawley (SD) rats (180-220 g) and mice (20-25 g) of either sex were obtained from the animal house of the Aga Khan University, Karachi. The animals were housed in constant room temperature (23-25°C), kept in plastic cages (47 × 34 × 18 cm^3^) with sawdust (renewed after every 48 h) and had free access to food and water. Rats were starved for 16 hrs prior to experiment, anaesthetized with diethyl ether by inhalation and blood was collected via cardiac puncture. Experiments performed with the rulings of the Institute of Laboratory Animal Resources, Commission on Life Sciences [14].

### Preparation of diet

The following two types of diets were prepared.

1. **Normal diet**: The normal diet was prepared at the animal house of the Aga Khan University (AKU), Karachi. The standard diet consists of flour (5 kg), chokar (5 kg), salt (75 g), nutrivet L (33 g), molasses (150 g), potassium meta bisulphate (15 g), fish meal (2.25 kg), powdered milk (2 kg) and oil (500 g) for a total of about 15 kg of the food material.

2. **Atherogenic diet**: Cholesterol (2% w/w), cholic acid (0.5% w/w) and butter fat (5% w/w) were added to normal diet, as described by Ichihashi [15] with slight modification.

All measures were taken to ensure the uniform mixing of additives in dry ingredients of the diet before kneading.

### Triton WR 1339-induced model of hyperlipidemia

The tyloxapol-induced hyperlipidemic model earlier described by Khanna [16] was followed with slight modifications. Male SD rats weighing 180-220 g caged in uniform hygienic conditions and kept on standard pellet diet and water *ad libitum*. The rats were randomly divided into 8 groups, each containing 6 rats, 1) control group, 2) tritonized group (untreated) and 3-8) treated groups with plant materials (Mc.Cr.F, Mc.Cr.R and Mc.Cr.L). After 10 days of the treatment, all animals were fasted for 7 h and group 1 received saline (10 ml/kg; i.p.), while groups 2-8 were given tyloxapol (500 mg/kg; i.p.). On the next day, rats were anaesthetized with diethylether by inhalation and blood was collected via cardiac puncture for analysis of serum total cholesterol and triglycerides.

### High fat diet-induced model

The high fat diet-induced dyslipidemic model was used as earlier described [17] with slight modifications after pilot studies. The adult SD rats (180-220 g) were randomly divided into different groups, each containing 6 rats. Control group was given normal diet (served as normal control), untreated group given atherogenic diet (atherogenic control) and treated groups were given atherogenic diet plus drug administered orally. All the groups of animals had free access to water and diet. The diet consumption was monitored daily and the gain in body weight was monitored weekly. At the end of the treatment, rats were fasted for 16 hrs, anaesthetized with diethyl ether by inhalation, blood was collected via cardiac puncture and serum analyzed for lipid profile and glucose level.

### Biochemical studies

#### Estimation of lipid profile and glucose level

For the determination of serum total cholesterol, high density lipoprotein cholesterol, triglyceride and glucose, a methods described by the manufacture (Randox Laboratories Ltd., Co. Antrim, UK.) was used. For this method test sample (serum), standard and blank were pipetted using a micropipette in to eppendorf tube. The reaction mixtures were mixed well and incubated at 20- 25°C. After incubation, 0.25 ml of each reaction mixture was poured in 96 well plates and the absorbance was read at 490 nm against the reagent blank in micro plate reader (Model 680 Bio-Rad Laboratories UK Ltd). The concentration of absorbance was calculated from the slope of concentration curve of the standards.

#### Estimation of LDL-C, TC/HDL and atherogenic index

These result were calculated indirectly by using formula describe by [18].

LDL = TC - HDL - TG/5 and Atherogenic index= TC-HDL/HDL.

#### Acute toxicity

The test was performed as described earlier [18]. Animals were divided in different groups of 5 mice each and were administered increasing doses of the plant extracts (3, 5, 10 g/kg, p.o.), in 10 ml/kg volume. Another group of mice was administered saline (10 ml/kg, p.o.) served as a negative control. The mice were allowed food and water *ad libitum *during a 24 hr test period and kept under regular observation for gross behavioural changes and mortality.

#### Data analysis

All data were expressed as mean ± standard error of mean (SEM, n = number of experiments) and EC_50 _values are given as geometric mean with 95% confidence intervals (CI). One-way Analysis of Variance (one-way ANOVA) was used to compare the differences in means of more than two groups, followed by Tukey post-test to determine significant differences among the pairs. P-values less than 0.05 (p < 0.05) were considered as statistically significant. All the graphs, calculation and statistical analyses were performed using GraphPad Prism software version 4.00 for Windows (GraphPad Software, San Diego California USA, http://www.graphpad.com).

## Results

### Effect of *Morinda citrifolia *extracts on tyloxapol-induced hyperlipidemia

Administration of tyloxapol (triton WR-1339) caused a significant increase (p < 0.001) in serum total cholesterol and triglyceride of tritonized group as compared to animals in the control group. Pretreatment of the rats with Mc.Cr.F (1000 mg/kg), Mc.Cr.L (1000 mg/kg) and Mc.Cr.R (500 mg/kg) caused significant reduction in cholesterol and triglyceride level. The data are summarized in Table 1.

**Table 1:** Effect of *Morinda citrifolia *extracts on tyloxapol-induced hyperlipidemia

Groups	Total cholesterol (mg/dl)	Triglyceride (mg/dl)
Control	66.44 ± 4.84	94.36 ± 8.83
Tritonized	878.76 ± 15.20	5177.73 ± 318.92
Treatments		
Mc.Cr.F 1000 mg/kg/day	565.83 ± 40.72**	3457.30 ± 275.13*
Mc.Cr.F 500 mg/kg/day	777.11 ± 80.32	4647.44 ± 237.38
Mc.Cr.L 1000 mg/kg/day	620.32 ± 39.32*	3693.55 ± 70.52*
Mc.Cr.L 500 mg/kg/day	699.00 ± 25.87	4110.84 ± 338.59
Mc.Cr.R 500 mg/kg/day	540.69 ± 107.57**	2997.02 ± 669.2***
Mc.Cr.R 300 mg/kg/day	844.0091 ± 44.32	4849.91 ± 167.52

Vale shown are mean ± S.E.M of 6 determinations

One-way ANOVA followed by Tukey post-test

*P< 0.05, **P < 0.01 and ***P < 0.001 compared to atherogenic group.

### Effect of Fruit extract on high fat diet-induced dyslipidemia

The intake of atherogenic diet increased serum total cholesterol (TC), triglyceride, LDL-C, TC/HDL ratio, atherogenic index and glucose level as compared to the control group. The oral administration of fruit extract (1000 mg/kg) with atherogenic diet prevented the rise in serum TC, LDL-C, TC/HDL ratio and atherogenic index. The treatment with the fruit extract had no significant effect (p > 0.05) on the HDL-C and glucose levels as compared to the atherogenic group. However, there was no effect seen on body weight and daily diet consumption (p > 0.05). The data are summarized in Table 2.

**Table 2:** Effect of *Morinda citrifolia *fruit extract (1000 mg/kg) on high fat diet-induced hyperlipidemia

Parameters	Control	Atherogenic	Treated
Total cholesterol (mg/dl)	67.48 ± 7.25	438.50 ± 22.87	284.5 ± 51.7*
Triglyceride (mg/dl)	72.08 ± 5.21	107.42 ± 10.97	64.89 ± 5.85**
HDL (mg/dl)	40.78 ± 6.38	12. 65 ± 2.76	21.1 ± 2.9
LDL (mg/dl)	12.37 ± 9.44	404.39 ± 21.0	250.3 ± 52.2*
TC/HDL ratio	1.84 ± 0.38	39.6 7 ± 7.28	14.81 ± 3.5*
Atherogenic index	0.71 ± 0.16	26.31 ± 3.31	7.71 ± 1.6***
Glucose (mg/dl)	88.85 ± 5.32	155.18 ± 10.56	125.8 ± 12.9
Diet consumption g/day	155.4 ± 7.5	122.3 ± 8.4	128.3 ± 6.9
% of change in body weight	28.5 ± 2.6	55.9 ± 6.5	47.3 ± 4.1

Vale shown are mean ± S.E.M of 6 determinations

One-way ANOVA followed by Tukey post-test

*P < 0.05, **P < 0.01 and ***P < 0.001 compared to atherogenic group.

### Effect of leaves extract on high fat diet-induced dyslipidemia

The TC, triglyceride, LDL-C, TC/HDL ratio, atherogenic index and glucose levels of the atherogenic group were significantly increased as compared to the control group. Oral administration of leaves extract (1000 mg/kg) with atherogenic diet prevented the rise of serum TC, LDL-C, TC/HDL ratio, atherogenic index and glucose levels. The HDL-C was similar (p > 0.05) to that in the atherogenic group. The leaves extract significantly prevented the gain in average body weight and increased daily diet consumption as compared to the atherogenic group. The data are summarized in Table 3.

**Table 3:** Effect of *Morinda citrifolia *leaves extract (1000 mg/kg) on high fat diet induced hyperlipidemia

Parameters	Control	Atherogenic	Treated
Total cholesterol (mg/dl)	81.02 ± 10.1	446.63 ± 40.0	235.3 ± 22.3***
Triglyceride (mg/dl)	70.94 ± 5.2	107.3 ± 11.0	70.52 ± 11.0*
HDL (mg/dl)	39.98 ± 5.86	13.26 ± 3.14	17.85 ± 3.77
LDL (mg/dl)	25.85 ± 10.7	412.21 ± 40.3	203.38 ± 24.2**
TC/HDL ratio	2.55 ± 0.46	37.37 ± 8.56	16.51 ± 3.65*
Atherogenic index	1.17 ± 0.41	31.10 ± 6.23	15.51 ± 3.65*
Glucose (mg/dl)	83.88 ± 6.83	158.81 ± 10.13	102.91 ± 5.21*
Diet consumption g/day	150.91 ± 8.44	120.3 ± 6.41	140.8 ± 9.3**
% of change in body weight	25.04 ± 2.42	59.79 ± 3.68	26.50 ± 7.27***

Vale shown are mean ± S.E.M of 6 determinations

One-way ANOVA followed by Tukey post-test

*P < 0.05, **P < 0.01 and ***P < 0.001 compared to atherogenic group.

### Effect of root extract on high fat diet-induced dyslipidemia

The TC, triglyceride, LDL-C, TC/HDL ratio, atherogenic index and glucose levels in the atherogenic group were significantly increased as compared to the control group. Oral administration of root extract (500 mg/kg) with atherogenic diet prevented the rise in serum TC, LDL-C, TC/HDL ratio, atherogenic index and glucose levels. The treatment with (500 mg/kg) also increased HDL-C (p < 0.05) as compared to the atherogenic group. The root extract significantly prevented the gain in average body weight and increased daily diet consumption as compared to the atherogenic group (p < 0.001). The data are summarized in Table 4.

**Table 4:** Effect of *Morinda citrifolia *root extract (1000 mg/kg) on high fat diet induced hyperlipidemia

Parameters	Control	Atherogenic	Treated
Total cholesterol (mg/dl)	70.67 ± 5.76	384.81 ± 32.99	224.5 ± 26.6***
Triglyceride (mg/dl)	73.09 ± 5.21	109.42 ± 10.27	70.52 ± 10.99*
HDL (mg/dl)	43.34 ± 5.60	11.49 ± 3.26	43.82 ± 2.3***
LDL (mg/dl)	18.71 ± 5.21	351.44 ± 32.90	173.21 ± 2.0***
TC/HDL ratio	1.69 ± 0.15	52.01 ± 16.11	5.36 ± 0.83***
Atherogenic index	0.70 ± 0.2	24.41 ± 4.46	5.74 ± 2.11***
Glucose (mg/dl)	93.39 ± 5.4	157.67 ± 9.4	95.09 ± 9.0***
Diet consumption g/day	160.04 ± 5.5	118.3 ± 9.54	135.4 ± 8.7***
% of change in body weight	30.5 ± 2.63	55.19 ± 4.58	17.34 ± 3.4***

Vale shown are mean ± S.E.M of 6 determinations

One-way ANOVA followed by Tukey post-test

*P < 0.05 and ***P < 0.001 compared to atherogenic group.

### Safety study

The treatment with *Morinda citrifolia *fruit, leaves and root extracts for 10 day (tyloxapol study) and 6 weeks (high fat diet study) did not produce any death or behavioural changes in rats.

Acute toxicity testing (24 hour time) of Mc.Cr.F and Mc.Cr.L was conducted in mice at different doses (3, 5 and 10 g/kg) and there was no mortality or changes in gross behaviour observed up to the dose of as high as 10 g/Kg, as compared to control group. The roots extract (Mc.Cr.R) also did not cause any mortality and changes in gross behaviour up to the dose of 10 g/Kg as previously described [9].

## Discussion

*Morinda citrifolia *has been considered useful in cardiovascular diseases particularly hypertension, atherosclerosis and dyslipidemia. In this study we used different animal models to evaluate the possible mode of action(s) of antidyslipidemic effect of different parts of *Morinda citrifolia*. Tyloxapol is a non-ionic surfactant being widely used to explore possible mechanism of lipid lowering drugs, it causes drastic increase in serum triglycerides and cholesterol levels due to increase in hepatic cholesterol synthesis particularly by the increase in HMG Co-A (3-hydroxy-3-methyl-glutaryl Co-A) activity [19] and by the inhibition of lipoprotein lipase responsible for hydrolysis of plasma lipids [20]. In fasting condition the only source of serum lipid levels is the endogenous production. Significant inhibition of rise in lipid levels by extracts of various parts of *Morinda citrifolia *in this model is indicative of inhibition of cholesterol biosynthesis by inhibition of HMG Co-A. This enzyme plays a key role in controlling lipid levels in plasma and other tissue. Most of the newer antidyslipidemic drugs such as statins act via the inhibition of HMG Co-A. However, failure of the Noni extracts to cause complete inhibition indicates the involvement of additional mechanisms. *Morinda citrifolia *is reported to be rich in flavones [9,21,22], which are known to inhibit lipid biosynthesis [23]. High cholesterol diet induces endothelial dysfunction, atherosclerosis [24] and increases oxidative stress by increasing the expression of oxidation-sensitive genes, such as Elk-1 and p-CREB [25]. High cholesterol diet with cholic acid increases TC, LDL-C, atherogenic index and decrease HDL-C by enhancing intestinal absorption and secretion and decreasing catabolism of cholesterol [26]. Treatment with *Morinda citrifolia *extracts caused a significant decrease in mean serum TC and LDL-C while increased HDL-C. The plant extracts also caused significant reduction in the atherogenic index, which is considered a better indicator of coronary heart disease risk than individual lipoprotein concentration [27].

High fat diet also causes oxidative stress (enzymatic and non-enzymatic) in rats, thus, increases oxidation of low density lipoprotein (LDL) which plays key role in genesis of atherosclerosis. Antioxidants are known to effectively prevent this kind of damage [28]. The presence of strong antioxidant activities in various parts of *Morinda citrifolia *[29] may offer additional benefit in combating the oxidative stress caused by high cholesterol. Relatively strong antidyslipidemic activity in root extract may be due to the presence of more antioxidant activity in this part [10,29]. The active constituents responsible for antioxidant activity include 3,3'-bisdemethylpinoresinol, americanol A, morindolin, isoprincepin [30], scopoletin [31], kaempferol, ursolic acid, quercetin and various other constituents [30,32-34] [see additional file 1]

## Conclusions

The present study provides mechanisms of antidyslipidemic activities of various parts of *Morinda citrifolia *through multiple pathways i.e., inhibition of biosynthesis, absorption and secretion of lipids. This may be due to the presence of multiple potent antioxidant constituents in this plant, though additional mechanism(s) cannot be ruled out. The results from this study rationalize the medicinal use of *Morinda citrifolia *in dyslipidemia and it can be used as a potential medicine for cardiovascular diseases. However, further studies are required to prove the safety and efficacy of *Morinda citrifolia *and its constituents in actual clinical settings.

## Abbreviations

(MC.CR.F): *Morinda citrifolia *fruit extract; (MC.CR.L): *Morinda citrifolia *leaves extract; (MC.CR.R.): *Morinda citrifolia *root extract; (TC): total cholesterol; (TG): triglyceride; (LDL-C): low density lipoprotein-cholesterol; (HDL-C): high density lipoprotein-cholesterol; (SD): Sprague-Dawley.

## Competing interests

The authors declare that they have no competing interests.

## Authors' contributions

SRM carried out the experimental work, data collection and evaluation, literature search and draft preparation. AHG identified the plant, raised funds, supervised the work and refined the manuscript for publication. NA responsible for critical review, intellectual input in discussion and overall presentation of paper. All authors have read and approved the final manuscript.

## Supplementary Material

Additional file 1 **Chemical constituents from various parts of *Morinda Citrifolia***. Following list shows reported chemical constituents isolated from different parts of *Morinda citrifolia *plant. Click here for file
