# Supplementary material for: Studies on antidyslipidemic effects of Morinda citrifolia (Noni) fruit, leaves and root extracts
Source: Lipids Health Dis. 2010 Aug 20;9:88. doi: 10.1186/1476-511X-9-88 (PMC2939587; doi:10.1186/1476-511X-9-88)
Supplement: Additional file 1 — Chemical constituents from various parts of Morinda Citrifolia. Following list shows reported chemical constituents isolated from different parts of Morinda citrifolia plant. [file 1476-511X-9-88-S1.DOC]

**Additional File 1:**

**Title: Chemical constituents from various parts of *Morinda Citrifolia***

**Description:** Following list shows reported chemical constituents isolated from different parts of *Morinda citrifolia* plant. The relevant references are cited in the reference section of the manuscript.

***Fruit:*** 1, 3-dimethoxyanthraquinone, 1,2-dihydroxyanthraquinone, morinaphthalenone, hydroquinone, scopoletin, 1,8-dihydroxy-6-methoxy-3- methyl-9-anthrone, 2,4-dimethoxy-9-anthrone, morindafurone, morinaphthalene[33], 3,3‘-bisdemethylpinoresinol, americanol A, morindolin and isoprincepin[30].

***Leaves:*** The leaves are reported to contain stearic acid, palmitic acid, 5-benzofuran carboxylic acid -6-formyl methyl ester, 4-(3' (R)-hydroxybutyl)-3,5,5'-trimethyl-cyclohex-2-en-l-one, 4-hydroxy-4-[ 1 'E,3 'R)-3' -hydroxy-l 'butenyl]-3,5,5' -trimethyl-cyclohex-2-en-l-one [33], Citrifoside, 1,5,15-trimethylmorindol, Roseoside II, deacetyl asperuloside, kaempferol-3-O-a-L-rhamnopyranosyl-1(1→6)-b -D-glucopyranoside, ursolic acid, quercetin-3-O-a -L-rhamnopyranosyl-1(1→6)-b -D-glucopyranoside, 13-hydroxy-9,11,15-octadecatrienoic acid, pteryxin, peucedanocoumarin III, 5,15-dimethylmorindol (11),8) barbinervic acid, clethric acid, rotungenic acid, hederagenin, oleanolic acid, pheophorbide a, methyl pheophorbide b , methyl pheophorbide a, (S)-hydroxypurpurin-7 lactone dimethyl ester, (R)-hydroxypheophorbide a methyl ester, (R)-hydroxypurpurin-7 lactone dimethyl ester, (S)-hydroxypheophorbide a methyl ester, 3-Oacetylpomolic acid, 13-epi-phaeophorbide a methyl ester, and phytol [32]

***Roots:*** Deacetylasperulosidic acid, asperulosidic acid, damnacanthol-3-O-beta-D-primeveroside, lucidin 3-O-beta-D-primeveroside, morindone-6-O-beta-D-primeveroside [34].
